# Ostarine blunts the effect of endurance training on submaximal endurance in rats

**DOI:** 10.1007/s00210-024-03030-w

**Published:** 2024-03-07

**Authors:** Veselin Vasilev, Nikolay Boyadjiev, Petar Hrischev, Fanka Gerginska, Slavi Delchev, Desislava Arabadzhiyska, Marina Komrakova, Kai O. Boeker, Arndt F. Schilling, Katerina Georgieva

**Affiliations:** 1 Department of Physiology, Faculty of Medicine, Medical University of Plovdiv, 15-A “Vasil Aprilov” Blvd, Plovdiv, 4002 Bulgaria; 2 Department of Anatomy, Histology and Embryology, Faculty of Medicine, Medical University of Plovdiv, 15-A “Vasil Aprilov” Blvd, Plovdiv, 4002 Bulgaria; 3 Department of Clinical Laboratory, Faculty of Medicine, Medical University of Plovdiv, 15-A “Vasil Aprilov” Blvd, Plovdiv, 4002 Bulgaria; 4 Department of Trauma Surgery, Orthopaedics and Plastic Surgery, University Medical Center Goettingen, Robert-Koch-Str. 40, 37075 Goettingen, Germany

**Keywords:** Ostarine, SARMs, Endurance, Submaximal training, Testosterone

## Abstract

The purpose of this study is to study the effects of ostarine alone and in combination with endurance training in sexually mature, male Wistar rats. The rats were divided into a treadmill-trained group and a sedentary group. Half of each group received either ostarine or vehicle for 8 weeks (*n* = 10 each, in total *n* = 40). We examined some functional, hormonal, and anthropometric parameters and the myogenic gene expression of myostatin, insulin-like growth factor-1 (IGF-1), and vascular endothelial growth factor-A (VEGF-A) in m. gastrocnemius. Ostarine decreased submaximal endurance and increased myogenic gene expression of myostatin but had no effect on maximal time to exhaustion and grip strength. Training increased submaximal endurance, maximal time to exhaustion, and grip strength. Our results indicate that both exercise and ostarine treatment had no significant effects on serum levels of luteinizing hormone, follicle-stimulating hormone, and testosterone, or on the myogenic gene expression of IGF-1 and VEGF-A. Neither ostarine nor the training had a significant effect on the testis, liver, and heart weights. In conclusion, ostarine had no effect on anthropometric and hormonal parameters but increased the myostatin gene expression in muscle. The SARM treatment decreased submaximal endurance without affecting maximal time to exhaustion, and training increased both metrics.

## Introduction

Selective androgen receptor modulators (SARMs) are a class of molecules that interact with the androgen receptor. They can be divided into two groups: steroidal and non-steroidal. The steroidal types of SARMs are obtained by making structural changes to the testosterone molecule. Non-steroidal SARMs were discovered later and are grouped into different classes. The first class to be discovered was the arylpropionamides (Bhasin 2015; Narayanan et al. 2008). Nowadays, the non-steroidal group of modulators is more relevant, as its representatives exhibit weaker androgenic effects in addition to the pronounced anabolic effects known from the steroidal group. This allows a wide clinical application of the non-steroidal group as some of the side effects of anabolic androgenic steroids can be avoided. Non-steroidal SARMs could improve the therapeutic process in primary and secondary hypogonadism, osteoporosis, various forms of cachexia, and sarcopenia or be used for male contraception or as hormone replacement therapy (Gao and Dalton 2007). The most commonly used substances are ostarine, ligandrol, testolone, and andarine.

The first adverse effects associated with the use of non-steroidal SARMs were reported in 2010 (Geyer et al. 2014). Following an online survey of 343 non-steroidal SARM users, the most common adverse effects reported were reduced testicle size, acne outbreaks, and mood changes. Hair loss, irritability, and high blood pressure were rare. Most adverse effects occurred when SARMs were used for more than 3 months. The most common adverse effect with ostarine and testolone was mood changes (Efimenko et al. 2022).

Although there is still no approved drug from the group of non-steroidal androgen receptor modulators, they are freely available to anyone on the internet. They are available as bodybuilding products or as part of dietary supplements. However, users can easily be misled about the presence of non-steroidal SARMs in a supplement because they are sometimes referred to by different names (e.g., MK-2866 instead of ostarine) (Van Wagoner et al. 2017). This poses a risk of positive doping tests in professional athletes, as non-steroidal SARMs have been on the World Anti-Doping Agency Prohibited List since 2008 (Thevis and Schänzer 2018).

On the other hand, non-steroidal selective androgen receptor modulators represent a potential opportunity to improve the treatment of a large group of socially important diseases. By avoiding some of the side effects of anabolic steroids, they could improve patients’ quality of life. There is still insufficient data on their effects when used in combination with exercise. Non-steroidal SARMs are available to anyone and are used illegally by many amateurs to increase muscle mass and strength. Further studies are needed to establish some of the adverse side effects of using non-steroidal SARMs, as well as their effects in combination with exercise. In our experiment, we focused on the SARM ostarine (enobosarm, MK-2866, or GTx-024) because it is at the most advanced stage of clinical trials (Lambert 2021). We evaluated the effects of ostarine, training, and their combination on functional (grip strength, submaximal endurance, maximal time to exhaustion), hormonal (follicle-stimulating hormone (FSH), luteinizing hormone (LH), testosterone), anthropometric parameters (heart, liver, m. soleus weights, body mass index), and muscle gene expression of vascular endothelial growth factor-A (*Vegf-a*), insulin-like growth factor-1 (*Igf-1*), and myostatin (*Mstn*).

## Methods

### Experimental animals

In our experiment, we used sexually mature, male rats (*n* = 40) of the Wistar breed (from the vivarium of the Bulgarian Academy of Sciences). Their body weight was in the range of 160–200 g. The animals were kept in individual metabolic cages under the following conditions: 12/12 h light/dark cycle, temperature 22–24 °C, controlled humidity, and access to standard rat chow and water ad libitum. Body weight and food consumption were monitored weekly. The experimental protocol was approved by the Committee for Ethical Treatment of Animals at the Bulgarian Food Safety Agency (BFSA) (license no. 294) and the Committee for Scientific Ethics at the Plovdiv Medical University and was in accordance with the principles of the Declaration of Helsinki.

### Training regime

Because treadmill training is a skill that experimental animals must acquire, all rats ran on an EXER-3R Small Animal Treadmill (Columbus Instruments, Columbus, OH, USA) for 5 min daily, 3 days per week, at a treadmill speed of 25 m/min and incline of 5° for 2 weeks during the pre-experimental period. This duration of exercise does not induce adaptive changes (Lambert and Noakes 1989). Rats that did not learn to run on the treadmill were not included in the experimental groups. Forty spontaneously running rats were selected and divided into four experimental groups (*n* = 10 for each group): non-training group treated with vehicle (S + V), non-training group treated with ostarine (S + O), training group treated with vehicle (T + V), and training group treated with ostarine (T + O).

Training rats were subjected to 8 weeks of graded submaximal treadmill exercise. They ran at a treadmill speed of 25 m/min, 5° incline, 5 days a week. On the first training day, the exercise duration was 20 min, increasing by 5 min each day until a duration of 40 min was reached on the 5th training day. This exercise duration was maintained until the end of the experiment. To maintain motor skills for participation in the functional tests, the rats in the non-training groups also ran on a treadmill for 5 min, 3 days a week at the same speed and treadmill incline. One rat in the S + V group was removed from the experiment due to illness, and another rat in the T + V group was also removed due to injury and inability to complete the training protocol.

### Application of non-steroidal SARM

During the experimental period of 8 weeks, rats in the S + O and T + O groups were injected subcutaneously (sc) five times a week with 0.4 mg/kg ostarine dissolved in dimethyl sulfoxide (DMSO) (20% of the solution) and polyethylene glycol 300 (PEG 300) (80% of the solution). The SARM solution obtained after mixing with these solvents is stable for at least 1 year when stored at − 20 °C (Ventura et al. 2020). Ostarine (Shanghai Biochempartner Co., Ltd., China), dimethyl sulfoxide (Chimtex Ltd., Bulgaria), and PEG 300 (Merck, Germany) were used in the experiment. Animals in the other two groups receiving vehicle were injected only with PEG 300 and DMSO in the same volume sc. According to previous studies, the effects of SARMs in rats occur within the dose range from 0.04 to 4 mg/kg (Dalton et al. 2011). Based on this and similar to other studies, a median dose of 0.4 mg/kg was used in our study (Roch et al. 2020).

### Anthropometric parameters

The length of the animals was measured once at the end of the experiment, immediately before decapitation. The distance from the nose to the anus of each rat was measured (Wu-Peng et al. 1997). Abdominal girth, body mass index (BMI), and Lee index were also measured once at the end of the experiment, again immediately before decapitation. The following formula was used to calculate the BMI of the rats: BMI = body mass (g)/(nasoanal distance (cm))^2^ (Wu-Peng et al. 1997). To calculate the Lee index, the following formula was used: Lee index = body mass ^0.33^ (g)/(nasoanal distance (cm))^2^ (Peralta et al. 2002). Up to 3 min after decapitation of the rats, we determined the weight of the heart, liver, right m. soleus, and right testicle. A TP-512A laboratory balance (Denver Instrument, Germany) was used for this purpose.

### Submaximal endurance (SME)

At the beginning and end of the experiment, animals from all groups were subjected to the Lambert submaximal endurance test (Lambert and Noakes 1989; Georgieva and Boyadjiev 2004). Submaximal endurance was determined by running on a treadmill at a belt speed of 25 m/min and 5° inclination (which is about 70–75% of maximal oxygen consumption (VO_2max_)) (Georgieva et al. 2017). The time to fatigue and inability to maintain the rat’s position on the belt were recorded.

### Maximal time to exhaustion (MTE)

A maximum time to exhaustion (MTE) test was performed on all groups at the beginning and end of the experiment. A protocol of incrementally increasing exercise speed and treadmill belt inclination was used to achieve peak exercise. The protocol was as follows: step 1, 15 m/min, 5°; step 2, 19 m/min, 10°; step 3, 27 m/min, 10°; step 4, 27 m/min, 15°; step 5, 30 m/min, 15°; step 6, 35 m/min, 15°; and step 7, 40 m/min, 15°. The duration of each step was 3 min (Lambert and Noakes 1989; Georgieva and Boyadjiev 2004). The test was terminated when the rats were unable to maintain their position on the treadmill. The time taken for each rat to reach this state was taken as the maximum time to exhaustion.

### Grip strength

To measure the grip strength of the experimental animals, we used a grip strength meter (Ugo-Basile, Italy) connected to a force transducer (dynamometer) at the beginning and at the end of the experiment. Each rat was raised so that its forelimbs could grasp the test grid attached to the dynamometer. Each rat was then gently pulled backwards by its tail until it was released from the grid. The maximum force exerted by the rat before release was recorded by the apparatus and defined as grip strength. Three consecutive trials were performed on each rat, and as in other studies, the highest value was recorded as grip strength. The strength was expressed in grams (Tchekalarova et al. 2022).

### Hormonal parameters

Mixed blood was collected in monovets after decapitation of the experimental animals. Follicle-stimulating hormone (FSH), luteinizing hormone (LH), and testosterone concentrations were measured in serum using a Sirio Microplate Reader analyzer (SEAC, Italy). We used the ELISA method: sandwich enzyme immunoassay with two antibodies. Hormones from the samples formed a complex with an antibody fixed on the solid phase specific for rat hormones and a biotin-labeled polyclonal AT* (detecting antibody). After washing to remove unbound proteins, the AT-AG-AT* complex was bound to HRP-streptavidin (SABC). After incubation and washing to remove unbound HRP-streptavidin, the enzyme reaction was visualized using the substrate tetramethyl benzidine. The intensity of the colored product is proportional to the amount of the corresponding hormone in the sample. Duplicate samples were made. The kits used were from the company My BioSource (San Diego, CA, USA).

### Analysis of myogenic gene expression

The samples from m. gastrocnemius (*n* = 8 for each group) were homogenized in 750 µl TRIzol (Thermo Fischer Scientific, WA, USA) using 4-mm tungsten carbide beads (Cat. No. 69997 Qiagen, Germany) using the Tissuelyzer LT system (Qiagen, Germany). Samples were then incubated at room temperature for 5 min, followed by RNA extraction according to the manufacturer’s protocol (Trizol, Thermo Fisher Scientific) using chloroform and isopropanol treatments and an ethanol wash. Finally, the RNA pellet was dissolved in 20 µl H2O, measured using a DeNovix DS-11 FX + system (DeNovix, NC, USA), and stored at − 80 °C for further analysis. Reverse transcription was performed with 1000 ng total RNA using the iScript cDNA Synthesis Kit (Biorad, CA, USA). Real-time quantitative polymerase chain reaction (PCR) was performed using the detection marker SYBR Green (Biorad, CA, USA) and the CFX96 Real-time PCR Detection System (Biorad, CA, USA). The mRNA expression of Glycerinaldehyd-3-phosphat-Dehydrogenase (*Gapdh*), *Mstn*, *Igf-1*, and *Vegf-a* was measured in triplicate, and the effects were calculated using the 2-ΔΔCT method (Livak and Schmittgen 2001). Ready-to-use primers for *Gapdh*, *Igf-1*, and *Mstn* were ordered from Qiagen (QuantiTect Primer Assays, Qiagen, Hilden, Germany). For *Vegf-a*, the following primers were used: forward CTTGTTCAGAGCGGAGAAAGC and reverse ACATCTGCAAGTACGTTCGTT (Gordon et al. 2010).

### Statistical processing

Statistical analysis of the results was performed using SPSS v. 19.0. A two-way ANOVA was applied to assess the main effects of ostarine treatment (O) and submaximal endurance training (T) and the interaction between them. In the case of significant interactions between the factors, Tukey’s post hoc test was used to assess the differences between the groups. Body weight, MTE, and SME parameters were measured at the beginning and at the end of experiment (two time points), and the factor (time) was included in the analysis of variance. A paired *t*-test was used to analyze within-group differences in body weight, MTE, and SME between two time points. Differences at *p* < 0.05 were considered statistically significant. All data are presented as mean ± standard error of the mean (SEM).

## Results

### Effects on anthropometric parameters

Body weight in all experimental groups gradually increased over the course of the experiment. The effect of time was significant (*p* < 0.001 ANOVA), whereas the effects of ostarine treatment and exercise as well as their interactions did not significantly change the body weight (*p* > 0.05 ANOVA) (data have been published previously, Komrakova et al. 2023; Vasilev et al. 2024).

**Fig. 1:**
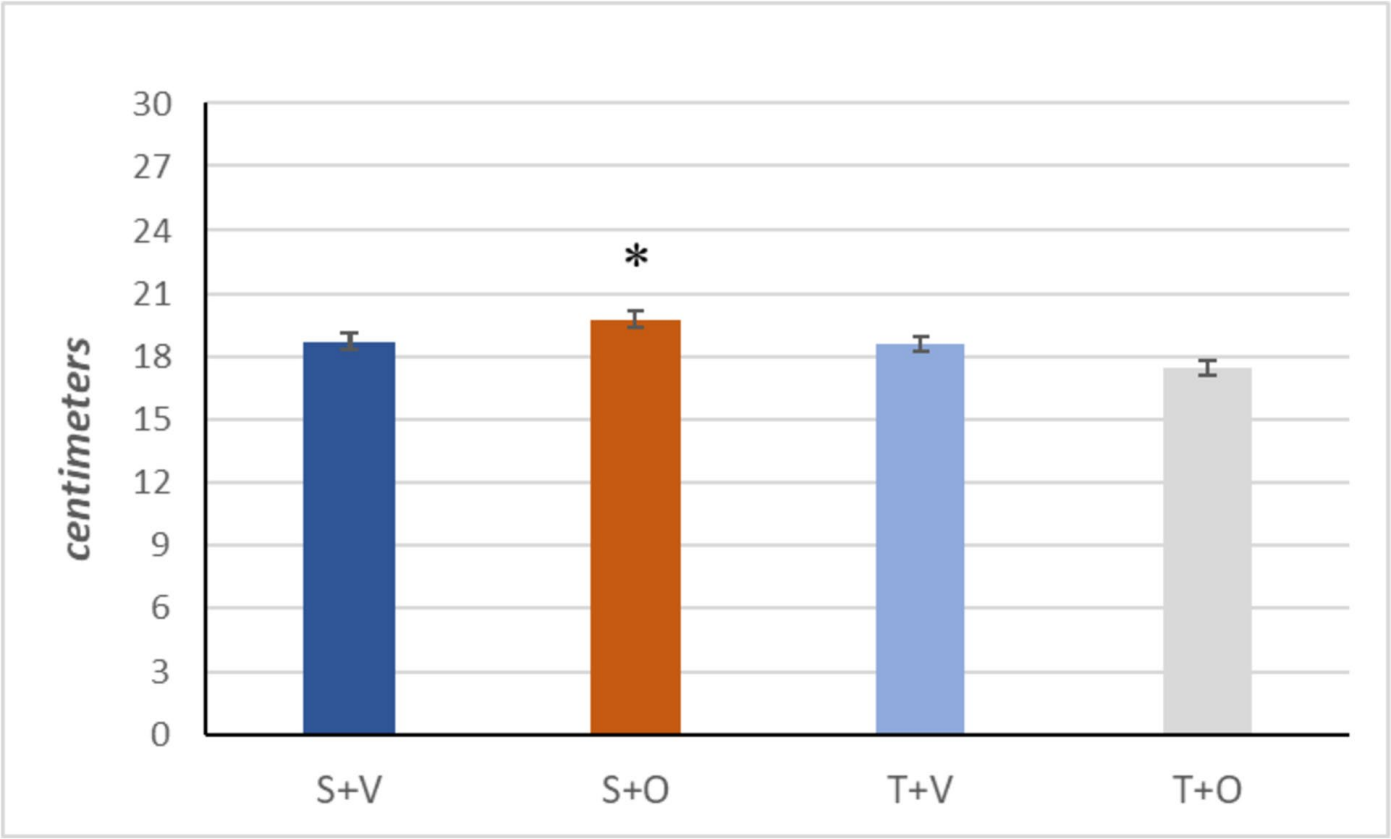
Abdominal girth of the experimental groups at the end of the experiment (mean ± SEM). Group S + V (*n* = 10); group S + O (*n* = 9); group T + V (*n* = 9); group T + O (*n* = 10); *p < 0.05 S + O compared to T + O at 8 week (Tukey post hoc test)

Statistical analysis revealed no significant main effect of ostarine treatment on the animals’ abdominal girth (*p* > 0.05 two-way ANOVA). Submaximal training had a significant effect on it, with the training groups having a smaller abdominal girth compared to the non-training group (*p* < 0.05, two-way ANOVA) (Table 1). There was a significant interaction between the two factors: The T + O group had a smaller abdominal girth than the S + O group (*p* = 0.014, Tukey post hoc test) (Fig. 1).

**Table 1:** Anthropometric indicators at the end of the experiment (mean ± SEM)

Variable	*Group*						
	T (*n* = 19)	S (*n* = 19)	V (*n* = 19)	O (*n* = 19)	T vs S	V vs O	T·O
Heart weight (g)	1.34 ± 0.04	1.33 ± 0.04	1.30 ± 0.04	1.37 ± 0.04	*p* = 0.818	*p* = 0.240	*p* = 0.175
Heart weight % of body weight (%)	0.31 ± 0.01	0.31 ± 0.01	0.30 ± 0.01	0.31 ± 0.01	*p* = 0.819	*p* = 0.328	*p* = 0.158
Liver weight (g)	12.69 ± 0.47	12.66 ± 0.47	12.56 ± 0.47	12.79 ± 0.47	*p* = 0.964	*p* = 0.735	*p* = 0.365
Liver weight % of body weight (%)	2.9 ± 0.06	3 ± 0.06	2.9 ± 0.06	3 ± 0.06	*p* = 0.658	*p* = 0.585	*p* = 0.083
Testis weight (g)	3.16 ± 0.12	3.23 ± 0.12	3.14 ± 0.12	3.26 ± 0.12	*p* = 0.707	*p* = 0.518	*p* = 0.902
M. soleus weight (g)	0.16 ± 0.08	0.13 ± 0.08	0.15 ± 0.08	0.13 ± 0.08	*p* = 0.014	*p* = 0.160	*p* = 0.123
Body length (centimeters)	24.2 ± 0.21	24 ± 0.21	24.3 ± 0.21	24.3 ± 0.21	*p* = 0.232	*p* = 0.974	*p* = 0.125
Abdominal circumference (cm)	19.2 ± 0.35	18.03 ± 0.35	18.65 ± 0.35	18.63 ± 0.35	*p* = 0.023	*p* = 0.918	*p* = 0.065
Lee index (g/cm^2^)	0.29 ± 0.003	0.30 ± 0.003	0.29 ± 0.003	0.30 ± 0.003	*p* = 0.140	*p* = 0.321	*p* = 0.456
BMI (g/cm^2^)	0.66 ± 0.02	0.66 ± 0.02	0.66 ± 0.02	0.65 ± 0.02	*p* = 0.952	*p* = 0.932	*p* = 0.620

*T*, training (groups T + V and T + O); *S*, sedentary (groups S + V and S + O); *V*, vehicle (groups S + V and T + V); *O*, ostarine (groups S + O and T + O); T vs S, main effect of training; V vs O, main effect of ostarine; T**·**O, interaction between the factors; (two-way ANOVA)

We found a significant main effect of endurance training, with trained rats having a greater weight of m. soleus compared to non-trained rats (*p* < 0.05 two-way ANOVA). Ostarine treatment had no significant main effect on soleus weight, and there was no significant interaction between the two factors (*p* > 0.05 two-way ANOVA) (Table 1).

Both endurance training and ostarine treatment had no significant effect (*p* > 0.05 two-way ANOVA) on heart and liver weights, BMI, and Lee index. We also found no significant differences (*p* > 0.05 two-way ANOVA) in the heart/body weight and liver/body weight ratios. For all the parameters listed, we found no significant interaction between the two factors (*p* > 0.05 two-way ANOVA) (Table 1).

### Effects on submaximal endurance

**Fig. 2:**
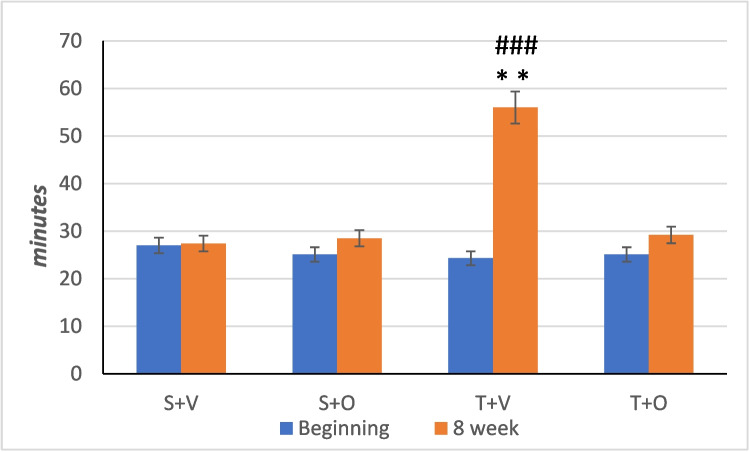
Submaximal running endurance of the experimental groups (mean ± SEM); group S + V (*n* = 10); group S + O (*n* = 9); group T + V (*n* = 9); group T + O (*n* = 10); **p < 0.01 in comparison with initial values of the same group (paired *t*-test); ###*p* < 0.001 T + V compared to every other group at 8 weeks (Tukey post hoc test)

In the SME study, the effects of “time,” “ostarine treatment,” and “exercise” as well as their interactions were highly significant (*p* < 0.001 ANOVA). There were no differences between the experimental groups at the beginning of the experiment (*p* > 0.05 two-way ANOVA). At the end of the experiment, only the T + V group had a significantly higher endurance compared to the baseline (*p* < 0.001 paired *t*-test) (Fig. 2).

We found a significant main effect of exercise, with the exercise groups having higher submaximal endurance than the non-training groups (*p* < 0.001 two-way ANOVA). The administration of ostarine also had a significant main effect, with the ostarine-treated groups having lower submaximal endurance than the placebo-treated groups (*p* < 0.001 two-way ANOVA) (Table 2).

**Table 2:** Functional and hormonal indicators and myogenic gene expression at the end of the experiment (mean ± SEM)

Variable	*Group*						
	T (*n* = 19)	S (*n* = 19)	V (*n* = 19)	O (*n* = 19)	T vs S	V vs O	T·O
SME (minutes)	42 ± 1.5	28 ± 1.5	41 ± 1.5	28 ± 1.5	*p* < 0.001	*p* < 0.001	*p* < 0.001
MTE (seconds)	869 ± 19	675 ± 19	761 ± 19	783 ± 19	*p* < 0.001	*p* = 0.439	*p* = 0.068
Grip strength (g/100 g BW)	112 ± 3.3	102 ± 3.3	106 ± 3.3	108 ± 3.3	*p* = 0.038	*p* = 0.749	*p* = 0.138
LH (ml U/ml)	9.1 ± 0.4	9 ± 0.4	8.8 ± 0.4	9.2 ± 0.4	*p* = 0.962	*p* = 0.562	*p* = 0.511
FSH (ng/ml)	31.2 ± 3.8	37.4 ± 3.8	35 ± 3.8	33.7 ± 3.8	*p* = 0.254	*p* = 0.811	*p* = 0.824
Testosterone (ng/ml)	11.3 ± 1.4	10.9 ± 1.4	12.3 ± 1.4	9.9 ± 1.4	*p* = 0.814	*p* = 0.223	*p* = 0.738
Mstn myogenic expression	1.81 ± 0.24	1.51 ± 0.24	1.16 ± 0.24	2.16 ± 0.24	*p* = 0.388	*p* = 0.004	*p* = 0.328
Vegf-a myogenic expression	1.28 ± 0.05	1.18 ± 0.05	1.15 ± 0.05	1.25 ± 0.05	*p* = 0.183	*p* = 0.060	*p* = 0.144
Igf-1 myogenic expression	0.99 ± 0.09	1.16 ± 0.09	1 ± 0.09	1.1 ± 0.09	*p* = 0.176	*p* = 0.235	*p* = 0.534

*T*, training (groups T + V and T + O); *S*, sedentary (groups S + V and S + O); *V*, vehicle (groups S + V and T + V); *O*, ostarine (groups S + O and T + O); T vs S, main effect of training; V vs O, main effect of ostarine; T**·**O, interaction between the factors; (two-way ANOVA)

There was also a significant interaction between the factors: the T + V group had a higher submaximal endurance than the S + V, S + O, and T + O (*p* < 0.001 Tukey post hoc test) groups (Fig. 2).

### Effects on maximal time to exhaustion

In the MTE analysis, the effects of “time” and “exercise” and their interaction were significant (*p* < 0.001 ANOVA), whereas the effect of “ostarine treatment” and its interaction with time and exercise were not significant (*p* > 0.05 ANOVA) (Table 2).

**Fig. 3:**
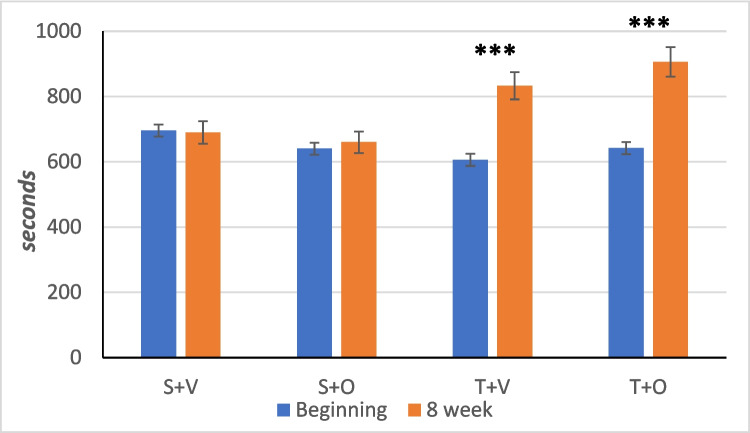
Maximum time to exhaustion of the experimental groups (mean ± SEM); group S + V (*n* = 10); group S + O (*n* = 9); group T + V (*n* = 9); group T + O (*n* = 10); ***p < 0.001—T + V and T + O in comparison with initial values of the same groups; (paired *t*-test)

Baseline values of maximum time to exhaustion were similar in all groups (*p* > 0.05 two-way ANOVA). At the end, the T + V and T + O groups significantly increased their time to exhaustion from baseline (*p* < 0.001 paired *t*-test) (Fig. 3).

Statistical analysis revealed a significant main effect of exercise, with the training groups having a higher maximum time to exhaustion compared to the non-training groups (*p* < 0.001 two-way ANOVA) (Table 2).

### Effects on grip strength

We found no differences between the groups at the beginning of the experiment when measuring grip strength (*p* > 0.05 two-way ANOVA, data not shown). At the end of the study, there was a significant main effect of exercise, with the training groups having greater grip strength than the non-training group (*p* < 0.05 two-way ANOVA). The 8 weeks of ostarine treatment had no significant main effect (*p* > 0.05 two-way ANOVA). There was no significant interaction between the factors (*p* > 0.05 two-way ANOVA) (Table 2).

### Effects on hormonal parameters

The administration of ostarine and submaximal training (*p* > 0.05 two-way ANOVA) had no significant effect on the concentration of testosterone, LH, and FSH. No significant interaction between the factors was found (*p* > 0.05 two-way ANOVA) (Table 2).

### Effects on myogenic gene expression

We found a significant main effect of ostarine administration on the mRNA expression of *Mstn* in the m. gastrocnemius. Treated groups had higher expression compared to vehicle groups (*p* < 0.01 two-way ANOVA). Endurance training had no significant main effect on *Mstn* expression (*p* > 0.05 two-way ANOVA). There was no significant interaction between the two factors (*p* > 0.05 two-way ANOVA) (Table 2).

Neither ostarine treatment nor submaximal exercise (*p* > 0.05 two-way ANOVA) had a significant main effect on *Igf-1* and *Vegf-a* expression in m. gastrocnemius. No significant interaction between the factors was found (*p* > 0.05 two-way ANOVA) (Table 2).

## Discussion

In the present study, we report for the first time the effect of ostarine on submaximal endurance and maximal time to exhaustion. To our knowledge, there are no data on the effects of non-steroidal SARMs on these functional parameters. With regard to maximal time to exhaustion, ostarine showed an effect similar to that of nandrolone decanoate, which did not significantly alter this parameter in either trained or non-trained rats (Georgieva and Boyadjiev 2004). Endurance training significantly increased MTE, confirming the results of other studies (Georgieva et al. 2017). Our results clearly showed that ostarine decreased SME and training increased it. The observed significantly higher result in the SME test of group T + V compared to group T + O indicates that ostarine also neutralized the beneficial effect of training on SME. As there were no differences between the two non-training groups in the SME test, we can say that ostarine negative effect on SME occurs only in training rats. In contrast to ostarine, the administration of nandrolone decanoate in combination with submaximal exercise has been reported to enhance the effects of exercise and resulted in a greater increase in SME than the isolated effect of exercise alone (Georgieva and Boyadjiev 2004). Other studies have also reported different effects on SME and MTE when the two parameters were examined in treadmill-trained rats (Lambert and Noakes 1989; Davies et al. 1981). Nandrolone decanoate also exerted different effects on the same two functional indicators (Georgieva and Boyadjiev 2004). The difference could be due to the fact that the increase of SME and MTE depends on different mechanisms (Lambert and Noakes 1989). The exact mechanisms influenced by ostarine need to be further examined.

Endurance training is thought to increase SME primarily through its beneficial effects on muscle oxidative capacity, maximal oxygen consumption, running economy, rate of muscle glycogen depletion, and increased angiogenesis in working muscles (McArdle et al. 2010). In our experiment, we found that ostarine did not significantly alter maximal oxygen consumption (Vasilev et al. 2024). *Vegf* is a member of the platelet-derived growth factor family and is the major regulator of angiogenesis through its receptor on the endothelial cells (Sherbet 2011). The lack of effect of ostarine on the gene expression of *Vegf-a* in m. gastrocnemius suggests that the non-steroidal SARM does not alter this mechanism for angiogenesis in working muscles either.

A possible mechanism for the lack of training effect under ostarine on the submaximal endurance could be the increased expression of *Mstn* gene in the gastrocnemius muscle. Myostatin, also known as growth-differentiation factor-8, is a known inhibitor of muscle growth and belongs to the transforming growth factor-beta family. It is a hormone produced by muscle cells and has an autocrine or paracrine effect. It binds to type II activin receptors (ActRIIA and ActRIIB) (Joulia-Ekaza and Cabello 2006). Myostatin determines the volume of muscle mass by regulating the number of muscle fibers formed prenatally and their postnatal growth and has a catabolic effect on protein metabolism. Mice lacking the myostatin gene develop twice the size of normal muscles (Esposito et al. 2022; Lee 2012). The observed effect of ostarine on SME may be due to the non-steroidal SARM-induced increase in *Mstn* myogenic expression. Further research is needed to discover how ostarine affected *Mstn* gene expression. Interestingly, endurance training alone had no significant effect on *Mstn* expression, so there may be concurring pathways at play here. Other authors report that only high-intensity training increased *Vegf* expression in the cardiac muscle (Yazdanian et al. 2018). We can assume that the lack of effect of training on the gene expression in the gastrocnemius muscle in our study might be because we used submaximal intensity of training and not higher.

The administration of ostarine did not change testicular weight. A decrease in testicular size was self-reported in 22% of ostarine users (Efimenko et al. 2022). If the effect is only visible in one out of five users, this suggests that in our experiment, it would be expected in 1–2 animals, so the group size may not have been sufficiently large to statistically detect such differences. It is known that long-term use of AAS also causes testicular atrophy (Boyadjiev et al. 2000). We found that rats receiving ostarine had a 20% lower serum testosterone concentration than rats receiving placebo, but the difference was not statistically significant. Ostarine did not cause any significant changes in serum levels of LH and FSH. A study of another non-steroidal SARM—GSK2881078—in healthy adult males showed similar results to ours. A reduction in androgen binding protein (SHBG) and total testosterone was reported, but no reduction in free testosterone, FSH, and LH (Clark et al. 2017). Given the lack of effect on gonadotropic hormones, it can be assumed that the observed reduction in total testosterone by some non-steroidal SARMs is not due to suppression of the hypothalamic-pituitary–testicular axis. However, administration of another non-steroidal androgen receptor modulator (C-6) to healthy rats resulted in a decrease in gonadotropin-releasing hormone and serum testosterone levels after 2 weeks of treatment and suppression of spermatogenesis after week 10 (Chen et al. 2005). Further studies are needed to clarify the exact mechanisms of these effects. It is likely that the different chemical structure of the different classes of non-steroidal SARMs has an impact on their effects (Nyquist et al. 2021). With regard to anabolic steroids, their 3-week use has been found to reduce serum concentrations of LH, FSH, and testosterone (Christou et al. 2017).

Our results indicate that 8 weeks of ostarine administration does not significantly alter liver weight. Cases of induced hepatotoxicity have been reported following the use of various non-steroidal SARMs at doses several times higher than those effective in clinical trials. Most cases have been reported following the use of ostarine or testolone. In the clinical cases available to us, the duration of abuse varied from 2 to 24 weeks. Some of the reported cases come from Flores et al. and Koller et al. (Flores et al. 2020; Koller et al. 2021). In all patients, gradual recovery of impaired liver function and improvement in clinicochemical parameters were observed after discontinuation of SARMs and treatment. No deaths or serious complications were reported. In our experiment, ostarine also did not significantly increase heart weight. There is one reported case of acute myocarditis following the use of testolone for an unknown period of time (Padappayil et al. 2022). The effects of steroidal SARMs also known as anabolic–androgenic steroids (AAS), which are toxic to both the heart and the liver, are well known. AAS induce left ventricular hypertrophy, increase myocardial mass, and increase the risk of myocardial infarction and sudden cardiac death (Boyadjiev et al. 2010). Regarding the liver, AAS can cause hyperbilirubinemia, increase in liver enzymes, and development of hepatocellular tumors (Neri et al. 2011; Singh et al. 1996). Endurance training did not significantly affect liver and heart weights. In the submaximal training we use, the main type of muscle fibers used is the slow ones—type I fibers. These fibers are predominant in the soleus muscle (85% in rats), which could explain the increase in its weight with training. Ostarine had no significant effect on the weight of this muscle.

IGF-1 belongs to the group of insulin-like growth factors (insulin, IGF-1, IGF-2). It is produced in various tissues, but mainly in the liver under the action of growth hormone. IGF-1 has a primary role in promoting the differentiation and growth of skeletal muscle (Miyake et al. 2007). The effects of IGF-1 on myogenic cells include stimulation of myoblast replication, myogenic differentiation, and myotube hypertrophy (Florini and Magri 1989). According to our results, ostarine and exercise did not induce significant changes in its expression. In contrast, a significant increase in *Igf-1* mRNA and *Igf-1* protein expressions was observed in 12-month-old male mice after 4 weeks of aerobic exercise, resistance exercise, and whole-body vibration in the gastrocnemius muscle (Li et al. 2022). Other studies have reported an increase in serum IGF-1 levels after resistance training in older men and women (Vale et al. 2009; Arazi et al. 2021). The discrepancy between published data and our results could be explained by differences in experimental design and the fact that mRNA expression does not always correspond to protein accumulation (Vogel and and Marcotte 2012).

The observed reduction in abdominal girth in the T + O group compared to S + O indicates that the effect of submaximal training on this anthropometric parameter is independent of ostarine intake. A similar effect of exercise on abdominal girth has been reported in another study, but in humans (Mekary et al. 2015). Endurance training did not affect the length of the rats, their body mass index, or the Lee index, confirming the results of other studies using a training model (Punhagui et al. 2018). Ostarine also did not alter these three parameters.

To our knowledge, the present study examines for the first time the effects of non-steroidal SARMs combined with endurance training on functional and hormonal indicators and on myogenic gene expression. However, there are some limitations in the study. We used only one SARM—ostarine for 8 weeks. Longer studies with several non-steroidal representatives of SARMs could provide additional information about the effects of this group of molecules. Future studies can examine the dose-dependent effects of SARMs when combined with training on a wider range of clinically important parameters.

## Conclusion

In conclusion, our study shows that ostarine reduces submaximal endurance by neutralizing the beneficial effect of training. Ostarine increased the myogenic gene expression of myostatin, which is probably related to the negative effect of ostarine on SME. We also found that the non-steroidal SARM ostarine did not significantly alter maximal time to exhaustion, grip strength, and serum concentrations of LH, FSH, testosterone, and myogenic gene expression of VEGF-A and IGF-1. Its application did not affect the heart, liver, and testis weights and other anthropometric parameters studied. Endurance training increased SME, MTE, grip strength, and soleus weight but did not significantly affect hormonal parameters and myogenic gene expression. Further studies are needed to clarify other aspects of the influence of non-steroidal SARMs on the body’s physical work capacity and to investigate their adverse effects with long-term use.

## Data Availability

The author confirms that all data generated or analysed during this study are included in this published article.
